# Dynamic Scapular Movement Analysis: Is It Feasible and Reliable in Stroke Patients during Arm Elevation?

**DOI:** 10.1371/journal.pone.0079046

**Published:** 2013-11-11

**Authors:** Liesbet De Baets, Sara Van Deun, Kaat Desloovere, Ellen Jaspers

**Affiliations:** 1 Rehabilitation Research Center - Biomedical Research Institute, Universiteit Hasselt, Diepenbeek, Belgium; 2 Department of Rehabilitation Sciences, Katholieke Universiteit Leuven, Heverlee, Belgium; 3 Clinical Motion Analysis Laboratory, University Hospital Pellenberg, Pellenberg, Belgium; 4 Neural Control of Movement Lab, Department of Health Sciences and Technology, ETH Zurich, Switzerland; University of Cambridge, United Kingdom

## Abstract

Knowledge of three-dimensional scapular movements is essential to understand post-stroke shoulder pain. The goal of the present work is to determine the feasibility and the within and between session reliability of a movement protocol for three-dimensional scapular movement analysis in stroke patients with mild to moderate impairment, using an optoelectronic measurement system. Scapular kinematics of 10 stroke patients and 10 healthy controls was recorded on two occasions during active anteflexion and abduction from 0° to 60° and from 0° to 120°. All tasks were executed unilaterally and bilaterally. The protocol’s feasibility was first assessed, followed by within and between session reliability of scapular total range of motion (ROM), joint angles at start position and of angular waveforms. Additionally, measurement errors were calculated for all parameters. Results indicated that the protocol was generally feasible for this group of patients and assessors. Within session reliability was very good for all tasks. Between sessions, scapular angles at start position were measured reliably for most tasks, while scapular ROM was more reliable during the 120° tasks. In general, scapular angles showed higher reliability during anteflexion compared to abduction, especially for protraction. Scapular lateral rotations resulted in smallest measurement errors. This study indicates that scapular kinematics can be measured reliably and with precision within one measurement session. In case of multiple test sessions, further methodological optimization is required for this protocol to be suitable for clinical decision-making and evaluation of treatment efficacy.

## Introduction

Shoulder pain is a common and disabling complication after stroke, affecting one-third of the stroke patients in general [1]. Moreover, bicipital tenderness, supraspinatus tenderness, and a positive Neer impingement sign are described in 54%, 48% and 30% of the stroke patients, respectively [2]. These problems negatively affect functional arm recovery and thereby decrease daily life autonomy and quality of life [3]–[5].

**Figure 1:**
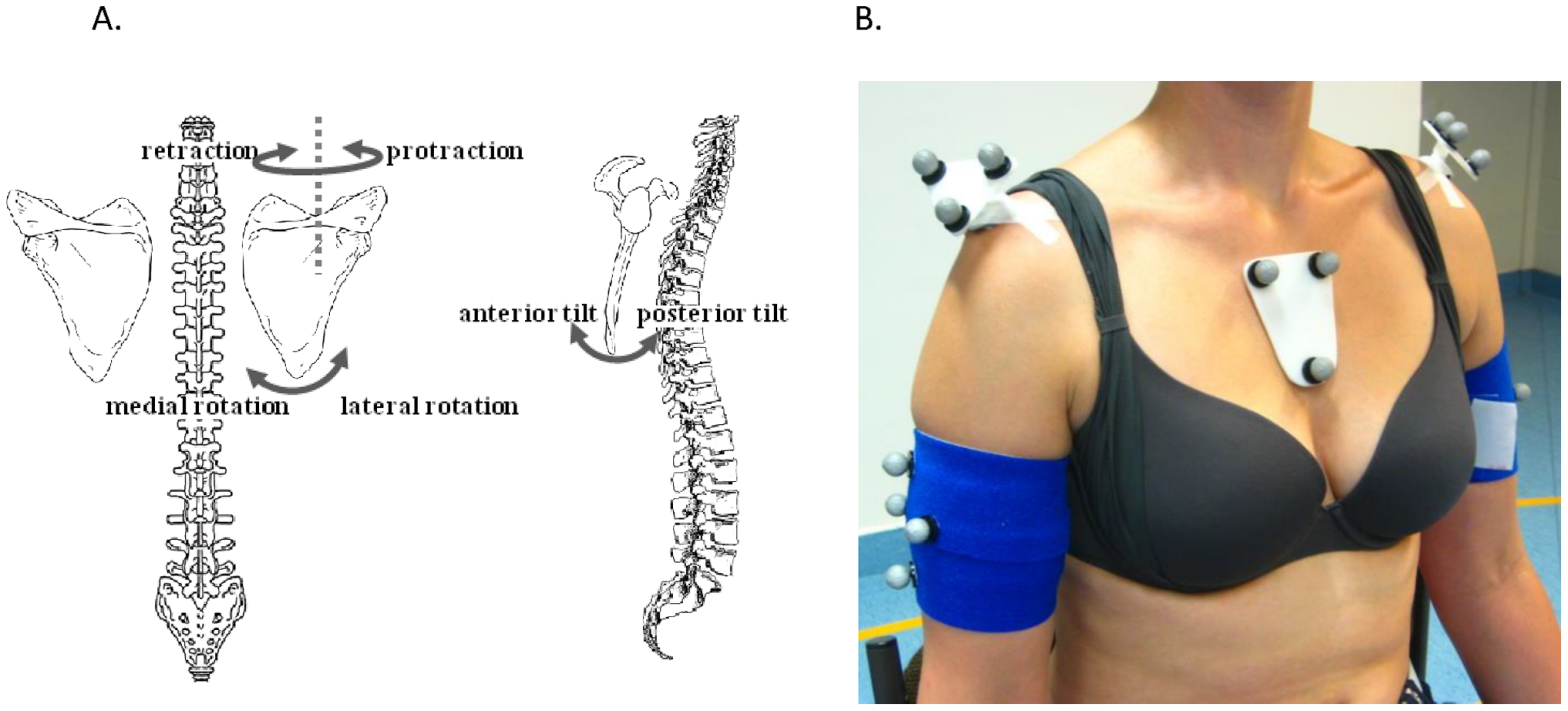
Overview of scapular rotations and marker placement. Three-dimensional scapular rotations (A) and marker cluster placement (B).

Careful assessment of the shoulder is thus essential in stroke patients. Motor scales used in routine clinical practice are typically limited to a global upper limb assessment [6]. Hence, key information on the isolated shoulder function and the more specific scapulothoracic movement is missed. However, given that adequate scapular behavior is a prerequisite for pain free shoulder movement, assessment of this scapulothoracic joint should be considered in stroke patients at risk to develop shoulder pathology and/or pain. Correct scapular movements are established by scapular muscles working in specific activation patterns [7]. Several neurological impairments (e.g. lack of muscle tone, spasticity and loss of motor control) will induce scapular muscular imbalances, which in turn will influence scapular position and movements. This altered scapular behavior is suggested to contribute to the development of rotator cuff impingement, and consequently to the development of shoulder pain [8]. Shoulder pathology has already been related to three-dimensional (3D) scapular movements (Figure 1A) during a humerothoracic elevation task. Despite the simplicity of this task, it has been shown sensitive enough to detect changes in 3D scapular movements associated with shoulder pathology [9]–[11]. More recently, measuring 3D scapular movements during humerothoracic elevation has also been introduced in stroke patients [12]–[15]. However, the value of such kinematic analysis in clinical decision-making or to evaluate treatment efficacy firstly requires the establishment of its feasibility and reliability [16].

The goal of the present work was to assess the feasibility and reliability of a specific humerothoracic elevation protocol to measure 3D scapular movements in stroke and in healthy controls. We furthermore aimed to formulate recommendations regarding parameter selection when using 3D scapular movements for clinical decision-making or to evaluate treatment efficacy.

## Methods

### Participants

An overview of the participants’ characteristics is given in Table 1. All stroke patients were hospitalized in the University Hospital Pellenberg and eligible for participation when they (1) were between 1 and 12 month(s) after a first time stroke; (2) had no shoulder complaints prior to stroke; (3) could perform 60° of humerothoracic elevation and (4) were able to understand the instructions. Stroke patients with an occipital, brainstem or cerebellar lesion or with reduced communicative or cognitive abilities were not considered for inclusion. Controls were recruited via family and colleagues and were excluded in case of current shoulder dysfunctions or treatment.

**Table 1:** Participants’ characteristics.

	Stroke patients	Healthy controls
Number of subject (men/women)	10 (7/3)	10 (4/6)
Age (years), range	18–69	18–70
Shoulder pain, yes/no	2/8	0/10
Hand dominance,left/right	0/10	0/10
Side of hemiplegia,left/right	2/8	NA
Time since stroke(weeks), range	5–39	NA
Lesion location	Cortical*	NA
Type of stroke, ischaemic/hemorhagic	7/3	NA
Fugl-Meyer score** (0–66), range	35–64	NA

* One patient had an addition lesion in the basal ganglia;

** Upper extremity motor section; NA: not applicable.

### Ethics Statement

This study was approved by the Ethical Committee of the University Hospital Pellenberg. All participants provided written informed consent to participate in this study, as approved by the Ethical Committee. The person on the photograph in Figure 1 and in Online Supplement 2 has given written informed consent (as outlined in PLOS consent form) to publish her photographs.

### Kinematic Data Acquisition

Bilateral 3D kinematic data were captured with 15 infrared cameras sampling at 100 Hz (Vicon, Oxford Metrics, UK) and filtered with spline-interpolation [17]. Clusters of three or four markers, mounted on tripods or cuffs, were placed on the sternum, scapula (flat part of the acromion) and upper arm (proximal, lateral) (Figure 1B). Anatomical landmarks were digitized during static trials, using a pointer with four linear markers. Anatomical landmarks were defined within their respective segmental marker cluster (CAST-procedure) [18], and subsequently used to construct anatomical coordinate systems and calculate joint kinematics. To ensure correct and accurate location of all anatomical landmarks, we adhered to specific palpation guidelines [19]. All kinematic calculations were done according to the ISB-guidelines [20]. Scapular kinematics were described for following three rotations: protraction/retraction, medial/lateral rotation, anterior/posterior tilting (Figure 1A).

### Measurement Procedure

Each participant was measured on two occasions, 5 to 10 days apart, by the same assessor. This assessor was trained to correctly conduct the measurement procedure and to perform the anatomical palpation properly. In this way, a repeatable and accurate placement of the marker clusters and palpation of anatomical landmarks was ensured. All measurements took place at the clinical motion analysis laboratory of the University Hospital Pellenberg. Marker clusters were mounted on the participant’s upper body, who was then seated on a chair with low back support. Next, static calibration trials were collected to digitize anatomical landmarks and participants were subsequently asked to execute the movement protocol (Table S1 and Figure S1): humerothoracic elevation in the frontal (abduction) and sagittal (anteflexion) plane, executed from 0° to 60° and from 0° to 120°. Each elevation task was done unilaterally and bilaterally at self-selected speed. Elevation height was marked on a pole to maximize standardization. Participants were given a practice trial prior to recording and each movement was demonstrated by an assessor seated in front of the participant. Three dynamic trials consisting of four repetitions each were recorded for every elevation task.

### Data Analysis

From the recorded trials, only the second and third repetition were selected for data analysis (as these were not corrupted by initiation/completion strategies), resulting in six cycles per elevation task per session. Movement cycles were visually defined from start to highest arm position. Data was further processed with Matlab®, using BodyMech (http://www.bodymech.nl) and custom-written routines. Each movement cycle was time-normalized and joint angles were visualized as function of time to check for erroneous signals. Discrete parameters of interest were (1) scapular range of motion (ROM) expressed for each scapular rotation in every elevation task, and (2) 3D scapular joint angles at start position. ROM was defined as the absolute difference between highest and lowest recorded joint angle per movement cycle.

### Statistical Analysis

Reliability of discrete parameters was calculated with the intraclass correlation coefficient (ICC) and the standard error of measurement (SEM) based on the square root of the mean square error term from the two-way ANOVA [16]. Single data from the first session was used to calculate within session reliability (ICC_w_(2,1); SEM_w_), averaged data from both sessions was used for between session reliability assessment (ICC_b_(2,k); SEM_b_). ICCs>0.80 were considered very high, 0.60–0.79 moderately high, 0.40–0.59 moderate and <0.40 low [21]. Percentage SEM (%SEM, i.e. (SEM/mean)*100) was additionally calculated for ROM [22] to indicate the preciseness [16] per rotation for each elevation task, relative to the total amount of ROM.

Within and between session reliability of angular waveforms was assessed with the adjusted coefficient of multiple correlation (CMC_w_;CMC_b_) [23] and group means were calculated. CMCs>0.90 were considered excellent, 0.80–0.89 good, 0.60–0.79 moderate and <0.60 poor. Waveform measurement errors (σ) were calculated and the ratio of between (σ_b_) to within session errors (σ_w_) was also reported [24].

## Results

**Figure 2:**
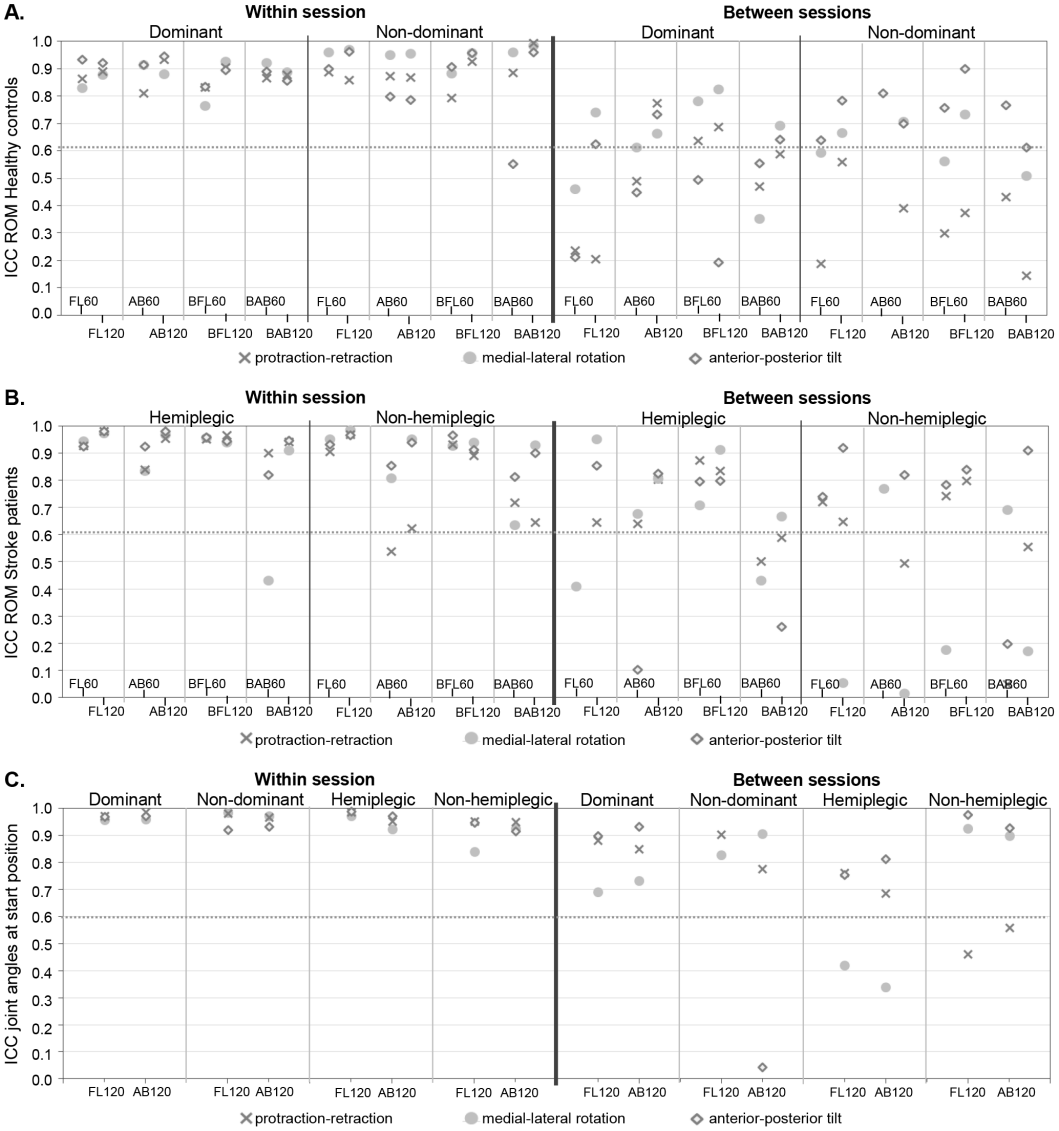
Within and between session reliability of parameters of interest. ICCs of range of motion in healthy controls (A), ICCs of range of motion in stroke patients (B) and ICCs of start position in healthy controls and stroke patients (C); FL: anteflexion; AB: abduction; BFL: bilateral anteflexion; BAB: bilateral abduction; No symbol is shown in case of calculation errors.

**Figure 3:**
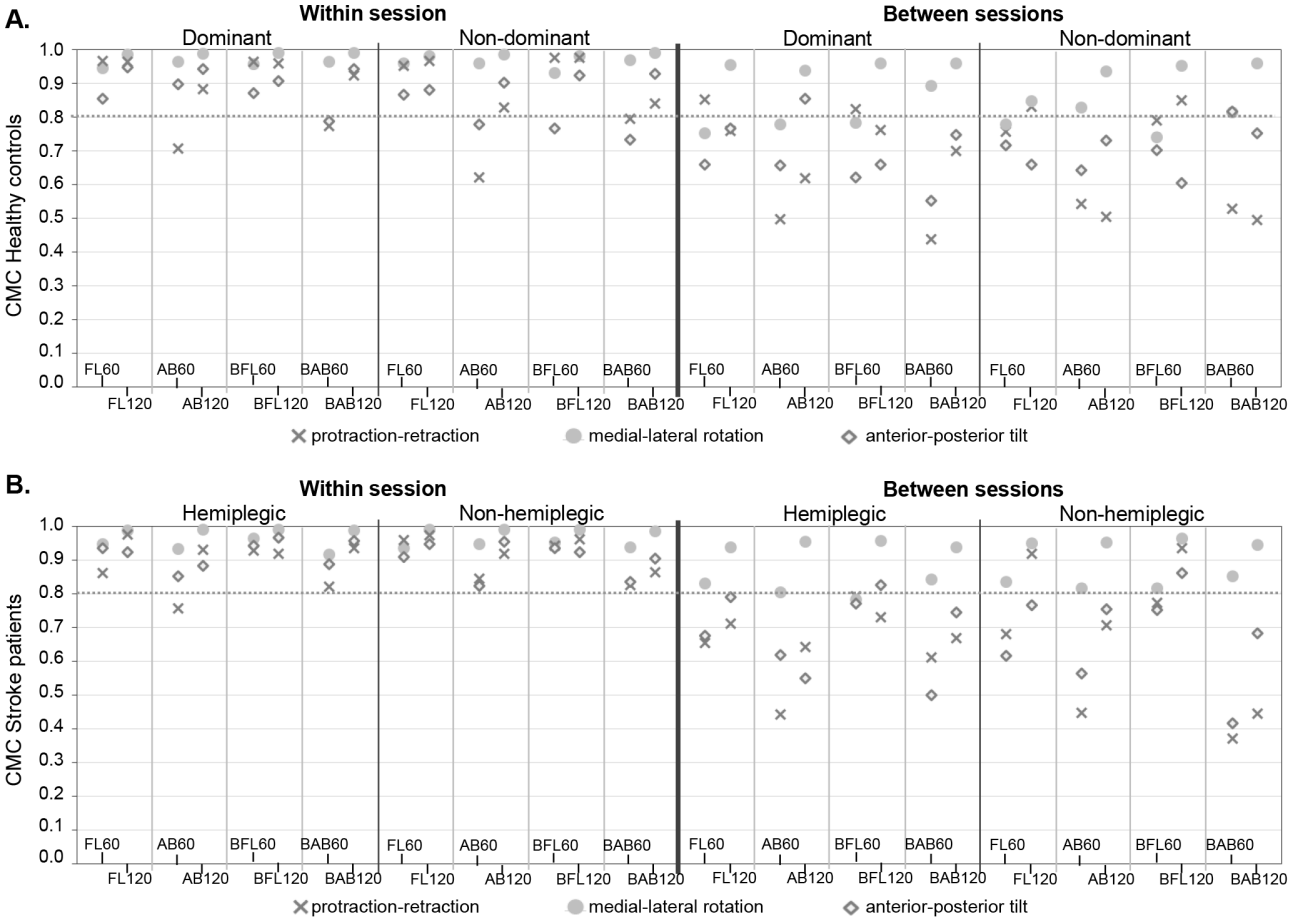
Within and between session reliability of angular waveforms. CMCs in healthy controls (A) and CMCs in stroke patients (B); FL: anteflexion; AB: abduction; BFL: bilateral anteflexion; BAB: bilateral abduction; No symbol is shown in case of calculation errors.

Figures 2 and 3 show within and between session ICCs and CMCs (see also Table S2 and S3) and measurement errors are listed in Table 2 and 3.

**Table 2:** Within session mean, standard deviation (SD) and standard error of measurement (SEM) for scapular range of motion (ROM).

	Anteflexion 60°				Anteflexion 120°				Abduction 60°					Abduction 120°			
	Mean	(SD)	SEM_w_	%SEM_w_	Mean	(SD)	SEM_w_	%SEM_w_	Mean	(SD)	SEM_w_		%SEM_w_	Mean	(SD)	SEM_w_	%SEM_w_
*Controls dominant side*																	
Protraction	13.2	(4.0)	1.5	**11.3**	17.1	(4.7)	2.3	**13.1**	4.4	(2.6)	1.0		21.7	13.8	(8.3)	1.8	**12.9**
Lateral rotation	10.6	(3.3)	2.1	19.4	37.6	(6.4)	3.4	**9.1**	13.4	(5.2)	1.8		**13.7**	41.0	(6.4)	3.6	**8.7**
Tilt	5.6	(3.1)	0.9	16.6	14.5	(4.9)	2.9	19.7	5.3	(3.8)	1.2		22.2	18.8	(9.1)	1.3	**7.1**
*Controls non-dominant side*																	
Protraction	14.0	(3.6)	1.3	**9.1**	18.9	(4.2)	2.1	**11.0**	5.3	(3.8)	1.7		31.1	12.5	(8.8)	5.2	41.6
Lateral rotation	15.9	(9.6)	2.0	**12.7**	42.5	(10.1)	1.9	**4.5**	18.6	(7.9)	1.7		**9.3**	44.8	(13.7)	6.1	13.6
Tilt	5.9	(3.3)	0.9	15.9	10.9	(6.7)	2.4	21.8	4.6	(2.3)	1.1		25.0	15.5	(6.8)	5.7	36.7
*Stroke hemiplegic side*																	
Protraction	12.0	(7.3)	3.6	30.1	20.8	(12.8)	1.1	**5.5**	6.3	(4.5)	1.4		22.7	15.2	(9.4)	1.1	**7.2**
Lateral rotation	17.6	(8.7)	2.6	**14.7**	43.0	(10.8)	1.9	**4.5**	20.1	(7.2)	4.3		21.6	47.2	(10.5)	2.5	**5.3**
Tilt	8.5	(4.1)	1.8	20.9	16.1	(10.4)	0.3	**2.1**	6.6	(4.8)	0.4		**6.0**	15.0	(11.8)	3.8	25.4
*Stroke non-hemiplegic side*																	
Protraction	14.2	(5.1)	2.5	17.6	21.4	(10.8)	0.9	**4.3**	5.4	(2.4)	1.8		33.8	12.4	(3.6)	1.2	**10.0**
Lateral rotation	15.4	(8.0)	2.1	**13.9**	45.5	(12.2)	1.4	**3.0**	17.0	(7.8)	6.1		36.0	48.5	(9.2)	4.1	**8.4**
Tilt	9.9	(5.2)	2.7	27.0	15.6	(9.0)	1.8	**11.5**	6.6	(4.4)	2.5		38.3	20.5	(8.8)	2.4	**11.7**
	**Bilateral anteflexion 60°**				**Bilateral anteflexion 120°**				**Bilateral abduction 60°**					**Bilateral abduction 120°**			
	**Mean**	**(SD)**	**SEM_w_**	**%SEM_w_**	**Mean**	**(SD)**	**SEM_w_**	**%SEM_w_**	**Mean**	**(SD)**		**SEM_w_**	**%SEM_w_**	**Mean**	**(SD)**	**SEM_w_**	**%SEM_w_**
*Controls dominant side*																	
Protraction	13.8	(3.8)	1.5	**10.9**	20.4	(5.8)	1.2	**6.0**	5.6	(3.8)		1.8	32.7	15.4	(8.4)	3.2	21.1
Lateral rotation	13.2	(3.9)	2.2	16.4	41.3	(7.6)	2.8	**6.9**	23.4	(10.0)		1.8	**7.6**	49.3	(7.3)	3.4	**6.9**
Tilt	5.0	(2.3)	0.7	**13.0**	13.1	(5.3)	2.3	17.2	5.4	(4.2)		0.7	**12.8**	18.0	(7.3)	4.9	27.1
*Controls non-dominant side*																	
Protraction	15.2	(3.7)	2.0	**13.5**	21.2	(5.9)	2.7	**12.5**	8.2	(5.5)		1.7	21.3	13.0	(7.9)	2.3	17.7
Lateral rotation	15.8	(7.4)	1.9	**12.0**	45.4	(8.7)	2.7	**6.0**	22.6	(8.9)		1.9	**8.3**	49.9	(14.7)	1.9	**3.8**
Tilt	6.8	(3.8)	0.8	**12.2**	12.7	(5.5)	1.0	**7.7**	5.4	(2.3)		1.3	24.5	13.6	(7.3)	1.2	**8.7**
*Stroke hemiplegic side*																	
Protraction	13.4	(8.5)	1.3	**9.8**	17.6	(9.1)	0.8	**4.4**	8.3	(6.7)		6.4	77.0	23.8	(14.9)	6.7	28.0
Lateral rotation	19.3	(8.4)	0.9	**4.9**	48.6	(8.9)	3.0	**6.1**	23.7	(7.7)		5.0	21.2	54.8	(8.0)	1.6	**2.9**
Tilt	8.9	(4.8)	1.8	20.2	17.7	(8.2)	2.4	**13.6**	6.5	(4.4)		1.9	29.6	24.5	(13.1)	3.8	15.7
*Stroke non-hemiplegic side*																	
Protraction	18.1	(8.4)	3.3	18.4	23.7	(9.5)	3.8	15.8	8.5	(4.1)		2.2	26.4	15.1	(5.1)	2.7	17.6
Lateral rotation	22.2	(9.0)	2.0	**9.2**	49.9	(11.2)	2.8	**5.6**	24.2	(7.7)		3.6	**14.9**	54.0	(11.1)	3.1	**5.7**
Tilt	10.5	(6.6)	1.2	**11.7**	16.1	(7.3)	1.5	**9.1**	7.5	(4.7)		3.1	41.4	16.9	(10.5)	2.5	**14.6**

Mean, SD and SEM are presented in degrees; %SEM represents the percentage SEM with respect to the mean; %SEMs lower than 15% are marked in bold.

**Table 3:** Between session mean, standard deviation (SD) and standard error of measurement (SEM) for scapular range of motion (ROM).

	Anteflexion 60°				Anteflexion 120°				Abduction 60°				Abduction 120°			
	Mean	(SD)	SEM_b_	%SEM_b_	Mean	(SD)	SEM_b_	%SEM_b_	Mean	(SD)	SEM_b_	%SEM_b_	Mean	(SD)	SEM_b_	%SEM_b_
*Controls dominant side*																
Protraction	13.5	(3.2)	3.1	23.0	17.8	(5.4)	5.4	30.3	4.1	(2.2)	1.9	47.3	11.8	(7.5)	3.7	31.5
Lateral rotation	11.0	(3.0)	2.6	23.8	37.1	(5.5)	3.9	**10.4**	14.1	(4.9)	3.8	26.9	40.5	(8.2)	6.4	15.7
Tilt	5.9	(3.5)	3.3	55.2	15.2	(6.8)	4.9	32.1	5.3	(3.4)	3.1	57.3	18.8	(9.1)	6.3	33.6
*Controls non-dominant side*																
Protraction	12.7	(3.9)	2.8	21.9	18.9	(5.8)	3.5	18.5	4.0	(2.9)	3.7	91.7	10.0	(7.0)	7.5	74.7
Lateral rotation	14.9	(7.4)	5.0	33.5	39.1	(10.3)	7.0	17.8	16.1	(6.7)	7.6	47.1	41.0	(13.0)	10.6	25.7
Tilt	6.2	(3.5)	2.7	43.0	11.7	(6.5)	4.6	39.5	4.8	(3.0)	2.3	48.1	14.7	(6.9)	5.0	34.0
*Stroke hemiplegic side*																
Protraction	10.9	(6.1)	6.1	56.2	19.7	(10.4)	3.4	17.2	4.7	(3.6)	0.4	**7.5**	13.3	(7.5)	4.7	36.1
Lateral rotation	16.8	(8.2)	6.2	36.9	42.3	(12.4)	5.0	**11.8**	20.4	(6.4)	3.9	19.1	46.0	(9.8)	5.4	**11.6**
Tilt	8.1	(4.0)	5.1	62.9	15.1	(8.5)	3.0	19.9	5.7	(4.1)	1.7	29.3	14.5	(10.3)	0.9	**6.3**
*Stroke non-hemiplegic side*																
Protraction	13.8	(4.6)	2.4	17.1	23.0	(9.2)	7.8	33.7	4.6	(3.0)	1.5	32.0	10.5	(3.5)	1.5	28.7
Lateral rotation	14.0	(7.2)	10.7	76.4	42.2	(11.2)	13.1	30.9	17.0	(6.8)	3.7	21.6	46.7	(10.0)	11.5	24.7
Tilt	9.6	(5.4)	2.8	29.1	14.6	(8.7)	4.3	29.6	6.0	(3.4)	1.8	30.2	18.2	(9.5)	5.8	31.7
	**Bilateral anteflexion 60°**				**Bilateral anteflexion 120°**				**Bilateral abduction 60°**				**Bilateral abduction 120°**			
	**Mean**	**(SD)**	**SEM_b_**	**%SEM_b_**	**Mean**	**(SD)**	**SEM_b_**	**%SEM_b_**	**Mean**	**(SD)**	**SEM_b_**	**%SEM_b_**	**Mean**	**(SD)**	**SEM_b_**	**%SEM_b_**
*Controls dominant side*																
Protraction	14.4	3.8	3.0	20.8	19.5	6.3	4.8	24.5	4.9	3.2	2.8	56.4	12.5	7.2	5.3	42.4
Lateral rotation	14.1	4.0	2.2	15.3	40.8	7.2	4.4	**10.8**	22.5	8.0	6.4	28.4	50.6	9.0	6.3	**12.5**
Tilt	6.1	3.3	2.9	46.8	13.3	6.2	5.3	39.8	5.3	3.8	2.7	50.2	16.0	6.8	4.9	30.4
*Controls non-dominant side*																
Protraction	13.9	3.4	1.6	**11.3**	20.9	7.0	5.7	27.2	6.8	4.3	4.3	63.2	11.6	7.8	7.3	62.9
Lateral rotation	16.4	7.0	5.1	31.3	43.2	9.6	6.8	15.7	20.7	7.4	8.7	40.3	48.5	11.9	10.0	20.6
Tilt	6.9	3.8	2.8	40.6	12.7	5.9	3.2	24.8	5.4	2.3	1.7	30.7	13.5	6.8	6.3	46.4
*Stroke hemiplegic side*																
Protraction	12.5	6.9	1.6	**12.5**	17.2	11.1	3.9	22.8	7.5	6.1	5.3	70.9	19.9	13.0	10.0	50.5
Lateral rotation	19.3	8.5	7.5	38.9	47.4	10.0	5.2	**10.9**	23.2	5.7	4.2	18.2	53.7	7.6	1.9	**3.6**
Tilt	8.9	4.3	1.9	20.9	15.8	7.6	3.3	20.9	5.3	3.5	1.8	33.6	20.4	12.8	7.1	34.8
*Stroke non-hemiplegic side*																
Protraction	16.8	6.9	5.1	30.5	25.6	8.1	5.2	20.2	6.7	3.7	3.0	44.3	12.0	4.7	3.7	30.4
Lateral rotation	21.3	7.9	10.7	50.3	48.6	8.7	10.8	22.3	23.8	8.1	8.0	33.8	52.4	11.8	12.4	23.7
Tilt	10.3	5.8	2.4	23.0	14.7	7.3	4.8	32.3	6.6	3.5	4.1	62.2	15.9	10.5	4.9	30.6

Mean, SD and SEM are presented in degrees; %SEM represents the percentage SEM with respect to the mean; %SEMs lower than 15% are marked in bold.

Three patients were not able to perform the 120° elevation tasks. Analysis of these tasks was therefore performed on seven patients.

### Range of Motion

Reliability results are given in Figure 2A and 2B, in Table 2 and 3, and in Table S2.

Within session reliability was moderately high to very high in patients and controls (ICCw 0.63–0.99), except for few scapular ROM during 60° abduction (non-dominant side tilt; hemiplegic side lateral rotation; non-hemiplegic side protraction).

Between session reliability for scapular ROM at the dominant side (controls) was in general moderately high to very highly reliable for lateral rotation and tilt for the 120° tasks (ICCb 0.63–0.83), protraction showed low to moderately high reliability results (ICCb 0.21–0.78). Results for the 60° tasks at the dominant side and for both the 60° and 120° tasks at the non-dominant side were less reliable. Patients showed more variable results between sessions. In general, all scapular ROM during the 120° tasks were moderately high to very highly reliable at the hemiplegic and non-hemiplegic side and lowest ICCb-values were found for lateral rotation at the non-hemiplegic side (ICCb 0.02–0.17). Reliability of scapular ROM during 60° elevation tasks was inconsistent in patients, with ICCs ranging from very low to very high for both sides (ICCb 0.05–0.88). In summary, we conclude for scapular ROM that 120° tasks are more reliable than 60° tasks, and that the dominant and hemiplegic side are slightly more reliably measureable than the non-dominant and non-hemiplegic side. No marked differences in reliability of scapular ROM were furthermore found for unilateral versus bilateral tasks.

%SEM-values depended not only on the task (60° vs. 120°) and side ((non)dominant vs. (non)hemiplegic), but also clearly differed for the three scapular rotations. In agreement with the ICCs, %SEM below 15% (higher precision) was found for the 120° tasks, especially at the dominant side (controls) and hemiplegic side (patients). In general, lateral rotation showed lower %SEM (<15% for all tasks within session and for 120° tasks at the dominant and hemiplegic side between sessions) than protraction (within sessions %SEM 4.3%–77%; between sessions %SEM 7.5%–91.7%) and tilt (within sessions %SEM 0.3%–41.4%; between sessions %SEM 6.3%–62.9%), especially between sessions.

### Joint Angles at Start Position

Only the results of joint angles at start for the following tasks are reported: unilateral 120° anteflexion and unilateral 120° abduction (Figure 2C, Table 4 and Table S2). Results for the other tasks were comparable.

**Table 4:** Within and between session mean, standard deviation (SD) and standard error of measurement (SEM) for scapular joint angles at start position.

	Within session						Between sessions					
	Anteflexion 120°			Abduction 120°			Anteflexion 120°			Abduction 120°		
	Mean	(SD)	SEM_w_	Mean	(SD)	SEM_w_	Mean	(SD)	SEM_b_	Mean	(SD)	SEM_b_
*Controls dominant side*												
Protraction	28.0	(8.0)	2.7	26.9	(8.5)	2.1	28.7	(7.0)	3.8	27.5	(7.5)	4.0
Lateral rotation	9.1	(7.0)	1.2	6.6	(7.2)	1.2	7.6	(6.5)	3.6	5.7	(6.9)	4.1
Tilt	8.0	(4.5)	0.6	8.5	(5.4)	0.4	7.3	(5.6)	2.8	8.1	(6.4)	2.7
*Controls non-dominant side*												
Protraction	26.6	(7.5)	0.9	27.0	(9.9)	2.3	25.9	(6.9)	3.5	24.9	(8.9)	3.5
Lateral rotation	6.9	(10.4)	1.9	3.2	(9.0)	2.2	4.1	(9.5)	3.5	4.0	(8.8)	3.6
Tilt	5.8	(3.4)	0.8	6.4	(4.5)	0.7	6.3	(4.9)	3.8	7.5	(5.5)	5.4
*Stroke hemiplegic side*												
Protraction	26.8	(8.0)	1.4	27.7	(6.4)	1.6	29.4	(8.1)	5.1	29.5	(6.6)	6.0
Lateral rotation	1.8	(6.3)	0.9	1.3	(4.6)	1.3	2.5	(6.8)	8.6	1.5	(6.1)	7.5
Tilt	10.3	(7.4)	1.1	9.0	(6.2)	1.9	9.2	(6.4)	4.5	9.7	(5.6)	3.9
*Stroke non-hemiplegic side*												
Protraction	34.6	(6.3)	1.9	32.7	(6.9)	2.0	32.1	(5.9)	3.9	30.7	(6.7)	5.6
Lateral rotation	0.8**^a^**	(2.9)	1.3	1.8**^a^**	(4.8)	1.6	0.8**^a^**	(2.8)	1.4	2.2**^a^**	(4.4)	1.4
Tilt	11.7	(5.1)	0.8	14.0	(4.4)	0.4	11.4	(5.0)	0.8	14.1	(4.4)	0.8

Mean, SD and SEM are presented in degrees; Bilat: Bilateral; **^a^**: Medial instead of lateral rotation.

Controls and patients showed very high within session reliability for all scapular rotations at start for both tasks (ICCw>0.84). Between session reliability of scapular angles at start was also moderately high to very high for both sides in controls (ICCb 0.69–0.93), apart from non-dominant side tilt (ICCb 0.04). In patients, highest between session reliability was found for tilt at start position of both sides and during both tasks (ICCb 0.75–0.98), followed by protraction at start (ICCb 0.46–0.76). For both tasks, start position of lateral rotation was also highly reliable at the non-hemiplegic side (ICCb>0.90), though was poorly reliable at the hemiplegic side (ICCb<0.45).

In patients and controls, within session SEM was below 3° for all scapular angles at start, for all tasks and at both sides; between session SEM was higher, with values ranging from 0.8° to 8.6°. Patients generally showed slightly higher SEMs at the hemiplegic side compared to the non-hemiplegic side and to controls.

### Angular Waveforms

Results are given in Figure 3, Table 5 and Table S3.

**Table 5:** Within and between session measurement errors of the angular waveforms (expressed in degrees) and the ratio of between to within errors.

	Anteflexion 60°			Anteflexion 120°			Abduction 60°			Abduction 120°		
	σ_w_	σ_b_	r	σ_w_	σ_b_	r	σ_w_	σ_b_	r	σ_w_	σ_b_	r
*Controls dominant side*												
Protraction	1.3	2.9	2.2	1.3	4.3	3.4	1.0	3.4	3.3	1.5	4.1	2.7
Lateral rotation	1.1	2.9	2.6	1.8	3.7	2.1	1.3	3.4	2.6	2.1	4.8	2.3
Tilt	0.9	2.1	2.4	1.0	2.8	2.8	0.9	2.3	2.7	1.5	3.4	2.3
*Controls non-dominant side*												
Protraction	1.1	4.1	3.7	1.3	5.2	4.1	0.9	4.6	4.9	1.8	5.3	2.9
Lateral rotation	1.1	3.8	3.4	1.9	7.4	3.8	1.6	3.8	2.4	2.4	5.1	2.1
Tilt	0.8	2.6	3.3	1.0	3.6	3.6	1.1	3.2	2.8	1.7	3.9	2.3
*Stroke hemiplegic side*												
Protraction	1.2	4.3	3.7	1.2	5.2	4.2	1.5	3.6	2.4	1.5	5.2	3.6
Lateral rotation	1.6	5.2	3.3	2.0	5.4	2.7	2.1	5.2	2.5	1.9	4.5	2.4
Tilt	0.9	2.5	2.8	1.3	2.6	2.0	1.1	2.2	2.0	1.5	3.2	2.1
*Stroke non-hemiplegic side*												
Protraction	1.3	5.1	3.8	1.4	4.5	3.3	1.0	4.2	4.3	1.4	4.0	2.9
Lateral rotation	1.5	2.6	1.8	1.9	4.6	2.5	1.9	3.2	1.6	3.0	4.3	1.4
Tilt	1.0	2.8	2.8	1.2	2.7	2.2	1.1	2.1	1.9	1.4	2.4	1.7
	**Bilat Anteflexion 60°**			**Bilat Anteflexion 120°**			**Bilat Abduction 60°**			**Bilat Abduction 120°**		
	**σ_w_**	**σ_b_**	**r**	**σ_w_**	**σ_b_**	**r**	**σ_w_**	**σ_b_**	**r**	**σ_w_**	**σ_b_**	**r**
*Controls dominant side*												
Protraction	1.2	3.4	2.9	1.6	4.6	2.9	1.1	3.8	3.5	1.7	4.4	2.5
Lateral rotation	1.3	3.0	2.4	1.8	3.6	2.0	1.7	3.7	2.1	2.1	4.8	2.2
Tilt	0.9	2.3	2.6	1.2	3.4	2.8	0.9	2.5	2.7	1.7	3.5	2.1
*Controls non-dominant side*												
Protraction	1.1	4.4	4.1	1.5	3.5	2.4	1.5	4.1	2.8	1.5	4.6	3.0
Lateral rotation	1.8	3.6	2.0	2.2	4.5	2.0	1.7	4.4	2.5	2.0	5.1	2.6
Tilt	1.2	2.6	2.2	1.1	3.4	3.2	1.1	3.4	3.1	1.2	3.9	3.1
*Stroke hemiplegic side*												
Protraction	1.2	3.6	2.9	1.3	5.1	4.0	2.0	5.4	2.7	1.8	6.8	3.8
Lateral rotation	1.4	4.9	3.4	2.0	4.8	2.4	3.3	4.7	1.4	2.7	5.9	2.2
Tilt	0.9	2.7	3.0	1.5	2.6	1.8	1.4	3.1	2.3	1.9	4.5	2.4
*Stroke non-hemiplegic side*												
Protraction	1.8	4.3	2.4	1.8	4.2	2.3	1.4	4.0	2.9	2.3	5.4	2.4
Lateral rotation	1.7	4.3	2.5	2.2	4.7	2.2	3.0	4.3	1.4	3.0	5.9	1.9
Tilt	1.0	2.3	2.3	1.6	2.3	1.4	1.4	2.4	1.7	2.2	3.4	1.6

σ_w_: within session error; σ_b_: between session error; r: ratio of σ_b_/σ_w;_ Bilat: Bilateral.

Within session reliability of angular waveforms was excellent for lateral rotation and moderate to excellent for protraction and tilt for all tasks and both sides in patients and controls. Between session reliability of angular waveforms was excellent for lateral rotation in 120° tasks and good to moderate in 60° tasks. For protraction and tilt, between session reliability ranged from poor to excellent, whereby higher values were found for anteflexion than abduction tasks.

Waveform measurement errors (σw-σb) were generally lower for anteflexion tasks compared to abduction tasks. Error ratio’s (σb/σw) ranged from 2.2 to 4.9 for protraction, from 1.4 to 3.8 for lateral rotation and from 1.4 to 3.6 for tilt.

## Discussion

This study investigated the feasibility and reliability of a protocol to measure 3D scapular kinematics. Such assessment is believed to provide additional information on the 3D character of scapular movements that is not captured with a two-dimensional analysis or the available clinical scales.

The discussion on feasibility of the applied methodology is twofold. Firstly, to ensure adequate assessment of scapular kinematics, patients should be able to perform the protocol as requested. Movements in the frontal plane were executed less accurately, especially by those patients with impaired arm proprioception, i.e. patients with more dysmetria during the Finger-to-Nose test of the Fugl-Meyer scale. As patients were instructed to look forward during task performance, they could not rely on visual feedback of their performance. Better guidance by means of e.g. a mirror or auditory signals is thus proposed. Furthermore, this protocol is specifically designed for stroke patients who are already relatively high functioning and thus at higher risk to develop shoulder pathology, and who would benefit most from scapular stabilization training in the prevention of or to treat e.g. shoulder pain. An active humerothoracic elevation of at least 60° is an absolute prerequisite to measure scapular behavior, and patients were selected accordingly in this study. The second feasibility issue focuses on the assessor. Although the use of the acromion marker cluster to measure scapular joint angles is validated by van Andel et al. (2009) [25], the assessor should be adequately trained to place the marker cluster and to perform the anatomical palpation in a repeatable manner. Therefore, a trained assessor with high knowledge in anatomical palpation performed all measurements in this study.

Reliability results of the current study showed that angles at start position were measured reliably in patients (hemiplegic side) and controls (dominant side) (ICC>0.60; SEM<8.6°), except lateral rotation at the hemiplegic side. As shoulder movement dysfunctions often find their origin in altered scapular start positions, these angles are highly relevant from a clinical viewpoint. The proposed method is thus a promising tool to measure the effect of scapular stabilization training. ROM was also generally more reliable at the dominant (controls) and hemiplegic arm (patients). For both groups, highest within session %SEMs were found for protraction in 60° abduction, suggesting a high natural intra-subject variability [24]. Since this variability cannot be controlled, 60° abduction tasks are considered less suitable to measure true changes in scapular protraction. Between sessions, tilt showed poorest ICCs and %SEMs, especially in 60° tasks. This indicates that a significant amount of methodological errors is introduced when measuring scapular tilt during 60° of sagittal or frontal plane elevation [24]. Hence, the effect of cluster placement on the acromion and palpation inaccuracies on scapular tilt should be further explored using advanced processing and analyzing techniques. Meskers et al. (2007) already proposed combining cluster recordings with recordings from a scapula locator at the beginning of every measurement [26]. This could serve as a check or correction for possible orientation changes of the cluster. ROM of scapular lateral rotation showed a high preciseness (lowest %SEM) within and between sessions, stressing the value of this angle in shoulder assessment, clinical decision-making and evaluation of treatment efficacy. The better %SEM-values for lateral rotation were however not reflected in overall higher ICCs. This inconsistency could be explained by the lower heterogeneity in results for lateral rotation, typically resulting in lower ICCs [16].

Apart from discrete joint angles, the similarity and measurement errors of angular waveforms were also assessed (CMCs and σ) [23], [24]. Higher CMCs were found for 120° elevation compared to 60° elevation, and anteflexion tasks showed better results than abduction tasks. Lateral rotation had highest CMCs for all tasks, in both patients and controls. For controls, lateral rotation was followed by protraction and tilting was least reliable during anteflexion. In contrast, tilting was more reliable than protraction during abduction. The scapula at the patients’ non-hemiplegic side performed similarly, while all rotations showed similar reliability at the hemiplegic side. The apparent differences in reliability between the three scapular rotations, based on the CMC, do not correspond to the waveforms’ measurement errors. Anteflexion did result in systematically lower waveform errors compared to abduction, in both patients and controls. This discrepancy can be explained by the inherent dependency of the CMC on the amplitude of the waveforms. Rotations with small amplitude typically result in lower CMCs. For instance, the 60° tasks optimally require a scapular setting, i.e. stabilization and not movement, resulting in small amplitudes and hence lower CMCs. Movement amplitudes for scapular lateral rotation are twice those of tilting and protraction, resulting in higher CMCs. Anteflexion also elicits more scapular protraction compared to abduction, again resulting in higher CMCs for protraction in the former task.

A summary of the major clinical implications according to these reliability results is given in Table 6.

**Table 6:** Implications for clinical use.

The proposed measurement protocol allows the reliable assessment of scapular angles at start position in healthy controls and stroke patients
120° tasks are most valuable for assessments of the full range of motion of scapular angles
Anteflexion tasks are more reliable compared to abduction tasks for discrete joint angles and waveforms, especially for protraction, and are thus preferred to use for clinical interpretation and decision-making
Measurement errors were lowest for lateral rotation, stressing the importance of this angle to assess i.e. treatment efficacy
Scapular angles at the patients’ hemiplegic arm and the controls’ dominant arm show slightly higher reliability, and should preferably be used in future

In literature, scapular joint angles during elevation have already been described in stroke patients. Meskers et al. [15] reported less protraction at the non-hemiplegic side compared to the dominant side of controls at different degrees of anteflexion. Niessen et al. [14] further found increased lateral rotation in rest at the non-hemiplegic side and during elevation at the hemiplegic side in stroke patients with shoulder pain compared to those without shoulder pain or controls. Conversely, Hardwick and Lang [12] described less lateral rotation at the hemiplegic side during active-assisted elevation. Comparable literature on the reliability of scapular kinematics in stroke patients is however scarce. The few available reliability studies in stroke [22], [27] did not report both within and between session measurement errors, thereby failing to discriminate natural variation from methodological errors. Moreover, the lack of consensus in applied methodology hinders proper result comparison. Van Andel et al. [25] applied a similar marker set-up and protocol in healthy young adults and reported somewhat worse ICCs and SEMs of scapular rotations at 120° elevation compared to those reported in this study. Additionally, Thigpen et al. [28] also reported highest CMCs during sagittal plane elevation for all scapular rotations in healthy adults. Roy et al. [29] reported slightly higher ICCs and SEMs compared to our results, though scapular angles were assessed during static arm positions in adults with and without impingement. Jaspers et al. [30] reported higher ICCs and lower SEMs in children with cerebral palsy during reaching tasks, which might be explained by the rigorous standardization of the test set-up and task execution.

The proposed measurement protocol allows the reliable assessment of scapular angles at start position in healthy controls and stroke patients. These angles are particularly interesting from a clinical viewpoint as arm movement dysfunctions often find their origin in altered scapular start positions. However, pain free shoulder movements additionally require adequate scapular behavior throughout task execution. The 120° tasks were most valuable for assessment of the full ROM of scapular angles. Whilst this restricts the protocol’s applicability, it also helps identifying those stroke patients at risk to develop shoulder pathology and/or pain. Anteflexion tasks also resulted in higher reliability compared to abduction tasks, especially for protraction, and are thus preferably used for clinical interpretation and decision-making. Furthermore, measurement errors were lowest for lateral rotation, stressing the importance of this angle to assess i.e. treatment efficacy. Scapular angles at the patients’ hemiplegic arm and the controls’ dominant arm were slightly better reliable, probably due to the reduced degrees of freedom in hemiplegic arms and the more controlled performance of dominant arms.

However, the results of this feasibility and reliability study should be interpreted with care due to the limited sample size, together with the stroke patients’ heterogeneity. Furthermore, this protocol is specifically designed for stroke patients with the ability to perform an active arm elevation. Therefore, this measurement method is limited to those patients with a moderate to mild motor impairment. A necessary future step in the analysis of scapular kinematics post stroke is the assessment of the discriminative ability of the proposed movement protocol. Factors such as age, pre-stroke hand dominance and time since stroke have been reported to impact on motor recovery and should therefore be taken into account [31], [32].

In conclusion, with the recommendations for task selection in mind, the measurement protocol is a valuable tool to assess scapular behavior and thereby contributes to the evaluation of arm dysfunction, the clinical decision-making and treatment planning in stroke patients.

## Supporting Information

Figure S1 **Start position and execution of the different elevation tasks.** Humerothoracic elevation (A) in the sagittal plane (anteflexion tasks) and (B) in the frontal plane (abduction tasks), executed from 0° to 60° and from 0° to 120°. Each elevation task was done unilaterally and bilaterally.
(DOCX)

Table S1 **Movement protocol: elevation tasks in order of performance.**
(DOC)

Table S2 **Intraclass correlation coefficients for scapular angle range of motion (ROM) and for start position of the 120° anteflexion and abduction tasks.**
(DOC)

Table S3 **The adjusted coefficient of multiple correlation for scapular waveforms.**
(DOC)
