# Supplementary material for: Dynamic Scapular Movement Analysis: Is It Feasible and Reliable in Stroke Patients during Arm Elevation?
Source: PLoS One. 2013 Nov 11;8(11):e79046. doi: 10.1371/journal.pone.0079046 (PMC3823991; doi:10.1371/journal.pone.0079046)
Supplement: Figure S1 — Start position and execution of the different elevation tasks. Humerothoracic elevation (A) in the sagittal plane (anteflexion tasks) and (B) in the frontal plane (abduction tasks), executed from 0° to 60° and from 0° to 120°. Each elevation task was done unilaterally and bilaterally. (DOCX) [file pone.0079046.s001.docx]

Figure S1. Start position and execution of the different elevation tasks

**B.**

**A..**

Humerothoracic elevation (A) in the sagittal plane (anteflexion tasks) and (B) in the frontal plane (abduction tasks), executed from 0° to 60° and from 0° to 120°. Each elevation task was done unilaterally and bilaterally.
