# Supplementary material for: Dynamic Scapular Movement Analysis: Is It Feasible and Reliable in Stroke Patients during Arm Elevation?
Source: PLoS One. 2013 Nov 11;8(11):e79046. doi: 10.1371/journal.pone.0079046 (PMC3823991; doi:10.1371/journal.pone.0079046)
Supplement: Table S1 — Movement protocol: elevation tasks in order of performance. (DOC) [file pone.0079046.s002.doc]

| Table S1. Movement protocol: elevation tasks in order of performance | | | |
| --- | --- | --- | --- |
|  | Side | | Elevation tasks |
|  | Healthy Controls | Stroke patients |  |
| Unilaterally | Dominant | Non-hemiplegic | Anteflexion 60° |
|  | Non-dominant | Hemiplegic | Anteflexion 60° |
|  | Dominant | Non-hemiplegic | Anteflexion 120° |
|  | Non-dominant | Hemiplegic | Anteflexion 120° |
|  | Dominant | Non-hemiplegic | Abduction 60° |
|  | Non-dominant | Hemiplegic | Abduction 60° |
|  | Dominant | Non-hemiplegic | Abduction 120° |
|  | Non-dominant | Hemiplegic | Abduction 120° |
| Bilaterally | Both sides | | Anteflexion 60° |
|  | Both sides | | Anteflexion 120 |
|  | Both sides | | Abduction 60° |
|  | Both sides | | Abduction 120° |
