# Supplementary material for: Dynamic Scapular Movement Analysis: Is It Feasible and Reliable in Stroke Patients during Arm Elevation?
Source: PLoS One. 2013 Nov 11;8(11):e79046. doi: 10.1371/journal.pone.0079046 (PMC3823991; doi:10.1371/journal.pone.0079046)
Supplement: Table S2 — Intraclass correlation coefficients for scapular angle range of motion (ROM) and for start position of the 120° anteflexion and abduction tasks. (DOC) [file pone.0079046.s003.doc]

| Table S2. Intraclass correlation coefficients for scapular angle range of motion (ROM) and for start position of the 120° anteflexion and abduction tasks. | | | | | | | | | | | | | | | | | | | | | | | | | | | | | | | | | | | | | | | | | | | | |
| --- | --- | --- | --- | --- | --- | --- | --- | --- | --- | --- | --- | --- | --- | --- | --- | --- | --- | --- | --- | --- | --- | --- | --- | --- | --- | --- | --- | --- | --- | --- | --- | --- | --- | --- | --- | --- | --- | --- | --- | --- | --- | --- | --- | --- |
|  | | Anteflexion 60° | | | | | | Anteflexion 120° | | | | | | | | | | | | | | | | Abduction 60° | | | | | | | | Abduction 120° | | | | | | | | | | | | |
|  | | ROM | | | | | | ROM | | | | | | | | Start position | | | | | | | | ROM | | | | | | | | ROM | | | | | | | | | Start position | | | |
|  | | ICC_w_ | | | ICC_b_ | | | ICC_w_ | | | | ICC_b_ | | | | ICC_w_ | | | | | ICC_b_ | | | ICC_w_ | | | | | | ICC_b_ | | ICC_w_ | | | | | ICC_b_ | | | | ICC_w_ | | ICC_b_ | |
| *Controls dominant side* | | | | | | | | | | | | | | | | | | | | | | | | | | | | | | | | | | | | | | | | | | | | |
| Protraction | | **0.87** | | | 0.24 | | | **0.89** | | | | 0.21 | | | | **0.97** | | | | | **0.88** | | | **0.81** | | | | | | 0.49 | | **0.94** | | | | | **0.78** | | | | **0.99** | | **0.85** | |
| Lateral rotation | | **0.83** | | | 0.46 | | | **0.88** | | | | **0.74** | | | | **0.96** | | | | | **0.69** | | | **0.92** | | | | | | **0.62** | | **0.88** | | | | | **0.67** | | | | **0.96** | | **0.73** | |
| Tilt | | **0.94** | | | 0.21 | | | **0.92** | | | | **0.63** | | | | **0.97** | | | | | **0.90** | | | **0.92** | | | | | | 0.45 | | **0.95** | | | | | **0.73** | | | | **0.97** | | **0.93** | |
| *Controls non-dominant side* | | | | | | | | | | | | | | | | | | | | | | | | | | | | | | | | | | | | | | | | | | | | |
| Protraction | | **0.89** | | | 0.19 | | | **0.86** | | | | 0.56 | | | | **0.98** | | | | | **0.90** | | | **0.87** | | | | | | Nan | | **0.87** | | | | | 0.39 | | | | **0.97** | | **0.78** | |
| Lateral rotation | | **0.96** | | | **0.60** | | | **0.97** | | | | **0.67** | | | | **0.99** | | | | | **0.83** | | | **0.95** | | | | | | Nan | | **0.96** | | | | | **0.71** | | | | **0.97** | | **0.91** | |
| Tilt | | **0.90** | | | **0.64** | | | **0.96** | | | | **0.79** | | | | **0.92** | | | | | Nan | | | **0.80** | | | | | | **0.81** | | **0.79** | | | | | **0.70** | | | | **0.93** | | 0.04 | |
| *Stroke hemiplegic side* | | | | | | | | | | | | | | | | | | | | | | | | | | | | | | | | | | | | | | | | | | | | |
| Protraction | | **0.93** | | | Nan | | | **0.98** | | | | **0.65** | | | | **0.99** | | | | | **0.76** | | | **0.84** | | | | | | **0.64** | | **0.96** | | | | | **0.80** | | | | **0.95** | | **0.69** | |
| Lateral rotation | | **0.95** | | | 0.41 | | | **0.98** | | | | **0.95** | | | | **0.97** | | | | | 0.42 | | | **0.84** | | | | | | **0.68** | | **0.97** | | | | | **0.81** | | | | **0.92** | | 0.34 | |
| Tilt | | **0.93** | | | Nan | | | **0.98** | | | | **0.86** | | | | **0.99** | | | | | **0.75** | | | **0.93** | | | | | | 0.11 | | **0.98** | | | | | **0.83** | | | | **0.97** | | **0.81** | |
| *Stroke non-hemiplegic side* | | | | | | | | | | | | | | | | | | | | | | | | | | | | | | | | | | | | | | | | | | | | |
| Protraction | | **0.91** | | | **0.72** | | | **0.97** | | | | **0.65** | | | | **0.95** | | | | | 0.46 | | | 0.54 | | | | | | Nan | | **0.63** | | | | | 0.50 | | | | **0.95** | | 0.56 | |
| Lateral rotation | | **0.95** | | | Nan | | | **0.99** | | | | 0.06 | | | | **0.84** | | | | | **0.93** | | | **0.81** | | | | | | **0.77** | | **0.95** | | | | | 0.02 | | | | **0.93** | | **0.90** | |
| Tilt | | **0.93** | | | **0.74** | | | **0.97** | | | | **0.92** | | | | **0.95** | | | | | **0.98** | | | **0.86** | | | | | | Nan | | **0.94** | | | | | **0.82** | | | | **0.92** | | **0.93** | |
|  | |  | | |  | | |  | | | |  | | | |  | | | | |  | | |  | | | | | |  | |  | | | | |  | | | |  | |  | |
|  | Bilat anteflexion 60° | | | | | | | | | Bilat anteflexion 120° | | | | | | | | | | | | | Bilat abduction 60° | | | | | | | | | | | Bilat abduction 120° | | | | | | | | | | |
|  | | ROM | | | | | | ROM | | | | | | | | | | | | | | | | ROM | | | | | | | | ROM | | | | | | | | | | | | |
|  |  | | ICC_w_ | | | ICC_b_ | | |  | | | | | ICC_w_ | | | ICC_b_ | | |  | | | | | | ICC_w_ | | | ICC_b_ | | | |  | | | | | ICC_w_ | | | | ICC_b_ | |  |
| *Controls dominant side* | | | | | | | | | | | | | | | | | | | | | | | | | | | | | | | | | | | | | | | | | | | | |
| Protraction | | **0.83** | | | **0.64** | | |  | | | | | **0.91** | | | | | **0.69** | | | |  | | | **0.87** | | | 0.47 | | | |  | | | | **0.88** | | | | 0.59 | | |  | |
| Lateral rotation | | **0.77** | | | **0.78** | | |  | | | | | **0.93** | | | | | **0.83** | | | |  | | | **0.92** | | | 0.35 | | | |  | | | | **0.89** | | | | **0.69** | | |  | |
| Tilt | | **0.84** | | | 0.50 | | |  | | | | | **0.90** | | | | | 0.20 | | | |  | | | **0.89** | | | 0.56 | | | |  | | | | **0.86** | | | | **0.64** | | |  | |
| ***Controls non-dominant side*** | | | | | | | | | | | | | | | | | | | | | | | | | | | | | | | | | | | | | | | | | | | | |
| Protraction | | **0.80** | | | 0.30 | | |  | | | | | **0.93** | | | | | 0.37 | | | |  | | | **0.89** | | | 0.43 | | | |  | | | | **0.99** | | | | 0.15 | | |  | |
| Lateral rotation | | **0.89** | | | 0.56 | | |  | | | | | **0.96** | | | | | **0.74** | | | |  | | | **0.96** | | | Nan | | | |  | | | | **0.99** | | | | 0.51 | | |  | |
| Tilt | | **0.91** | | | **0.76** | | |  | | | | | **0.96** | | | | | **0.90** | | | |  | | | 0.55 | | | **0.77** | | | |  | | | | **0.96** | | | | **0.62** | | |  | |
| ***Stroke hemiplegic side*** | | | | | | | | | | | | | | | | | | | | | | | | | | | | | | | | | | | | | | | | | | | | |
| Protraction | | **0.95** | | | **0.88** | | |  | | | | | **0.97** | | | | | **0.84** | | | |  | | | **0.90** | | | 0.50 | | | |  | | | | **0.94** | | | | 0.59 | | |  | |
| Lateral rotation | | **0.96** | | | **0.71** | | |  | | | | | **0.94** | | | | | **0.91** | | | |  | | | 0.43 | | | 0.43 | | | |  | | | | **0.91** | | | | **0.67** | | |  | |
| Tilt | | **0.96** | | | **0.80** | | |  | | | | | **0.95** | | | | | **0.80** | | | |  | | | **0.82** | | | Nan | | | |  | | | | **0.95** | | | | 0.26 | | |  | |
| ***Stroke non-hemiplegic side*** | | | | | | | | | | | | | | | | | | | | | | | | | | | | | | | | | | | | | | | | | | | | |
| Protraction | | **0.93** | | | **0.75** | | |  | | | | | **0.89** | | | | | **0.80** | | | |  | | | **0.72** | | | 0.05 | | | |  | | | | **0.65** | | | | 0.56 | | |  | |
| Lateral rotation | | **0.93** | | | 0.18 | | |  | | | | | **0.94** | | | | | Nan | | | |  | | | **0.64** | | | **0.69** | | | |  | | | | **0.93** | | | | 0.17 | | |  | |
| Tilt | | **0.97** | | | **0.79** | | |  | | | | | **0.91** | | | | | **0.84** | | | |  | | | **0.81** | | | 0.20 | | | |  | | | | **0.90** | | | | **0.91** | | |  | |
|  | |  | |  | | |  | | | |  | | | |  | | | |  | | | |  | | | |  | | | |  | | | |  | | | |  | | | |  | |
| ICC_w_: Intraclass correlation coefficients within session; ICC_b_: Intraclass correlation coefficients between sessions; Bilat: Bilateral; Nan: Could not be calculated; ICCs higher than 0.60 are marked in bold | | | | | | | | | | | | | | | | | | | | | | | | | | | | | | | | | | | | | | | | | | | | |
|  | |  | |  | | |  | | | |  | | | |  | | | |  | | | |  | | | |  | | | |  | | | |  | | | |  | | | |  | |
