# Supplementary material for: Dynamic Scapular Movement Analysis: Is It Feasible and Reliable in Stroke Patients during Arm Elevation?
Source: PLoS One. 2013 Nov 11;8(11):e79046. doi: 10.1371/journal.pone.0079046 (PMC3823991; doi:10.1371/journal.pone.0079046)
Supplement: Table S3 — The adjusted coefficient of multiple correlation for scapular waveforms. (DOC) [file pone.0079046.s004.doc]

| Table S3. The adjusted coefficient of multiple correlation for scapular waveforms. | | | | | | | | | | | | | | | | | | | | | | | | | | | | | | | | | | | | | | | | | | | | |
| --- | --- | --- | --- | --- | --- | --- | --- | --- | --- | --- | --- | --- | --- | --- | --- | --- | --- | --- | --- | --- | --- | --- | --- | --- | --- | --- | --- | --- | --- | --- | --- | --- | --- | --- | --- | --- | --- | --- | --- | --- | --- | --- | --- | --- |
|  | | Anteflexion 60° | | | | | | Anteflexion 120° | | | | | | | | | | | | | | | | Abduction 60° | | | | | | | | Abduction 120° | | | | | | | | | | | | |
|  | | CMC_w_ | | | CMC_b_ | | |  | | | | CMC_w_ | | | | CMC_b_ | | | | |  | | | CMC_w_ | | | | | | CMC_b_ | |  | | | | | CMC_w_ | | | | CMC_b_ | |  | |
| *Controls dominant side* | | | | | | | | | | | | | | | | | | | | | | | | | | | | | | | | | | | | | | | | | | | | |
| Protraction | | **0.97** | | | **0.85** | | |  | | | | **0.97** | | | | **0.76** | | | | |  | | | **0.71** | | | | | | 0.50 | |  | | | | | **0.89** | | | | **0.62** | |  | |
| Lateral rotation | | **0.95** | | | **0.75** | | |  | | | | **0.99** | | | | **0.96** | | | | |  | | | **0.96** | | | | | | **0.78** | |  | | | | | **0.99** | | | | **0.94** | |  | |
| Tilt | | **0.86** | | | **0.66** | | |  | | | | **0.95** | | | | **0.77** | | | | |  | | | **0.90** | | | | | | **0.66** | |  | | | | | **0.94** | | | | **0.86** | |  | |
| *Controls non-dominant side* | | | | | | | | | | | | | | | | | | | | | | | | | | | | | | | | | | | | | | | | | | | | |
| Protraction | | **0.95** | | | **0.76** | | |  | | | | **0.97** | | | | **0.83** | | | | |  | | | **0.62** | | | | | | 0.54 | |  | | | | | **0.83** | | | | 0.51 | |  | |
| Lateral rotation | | **0.96** | | | **0.78** | | |  | | | | **0.98** | | | | **0.85** | | | | |  | | | **0.96** | | | | | | **0.83** | |  | | | | | **0.99** | | | | **0.94** | |  | |
| Tilt | | **0.87** | | | **0.72** | | |  | | | | **0.88** | | | | **0.66** | | | | |  | | | **0.78** | | | | | | **0.64** | |  | | | | | **0.90** | | | | **0.73** | |  | |
| *Stroke hemiplegic side* | | | | | | | | | | | | | | | | | | | | | | | | | | | | | | | | | | | | | | | | | | | | |
| Protraction | | **0.86** | | | **0.66** | | |  | | | | **0.98** | | | | **0.71** | | | | |  | | | **0.76** | | | | | | 0.44 | |  | | | | | **0.93** | | | | **0.65** | |  | |
| Lateral rotation | | **0.95** | | | **0.83** | | |  | | | | **0.99** | | | | **0.94** | | | | |  | | | **0.94** | | | | | | **0.80** | |  | | | | | **0.99** | | | | **0.96** | |  | |
| Tilt | | **0.94** | | | **0.68** | | |  | | | | **0.93** | | | | **0.79** | | | | |  | | | **0.85** | | | | | | **0.62** | |  | | | | | **0.89** | | | | 0.55 | |  | |
| *Stroke non-hemiplegic side* | | | | | | | | | | | | | | | | | | | | | | | | | | | | | | | | | | | | | | | | | | | | |
| Protraction | | **0.96** | | | **0.68** | | |  | | | | **0.97** | | | | **0.92** | | | | |  | | | **0.85** | | | | | | 0.45 | |  | | | | | **0.92** | | | | **0.71** | |  | |
| Lateral rotation | | **0.94** | | | **0.84** | | |  | | | | **0.99** | | | | **0.95** | | | | |  | | | **0.95** | | | | | | **0.82** | |  | | | | | **0.99** | | | | **0.95** | |  | |
| Tilt | | **0.91** | | | **0.62** | | |  | | | | **0.95** | | | | **0.77** | | | | |  | | | **0.83** | | | | | | 0.57 | |  | | | | | **0.96** | | | | **0.75** | |  | |
|  | |  | | |  | | |  | | | |  | | | |  | | | | |  | | |  | | | | | |  | |  | | | | |  | | | |  | |  | |
|  | Bilat anteflexion 60° | | | | | | | | | Bilat anteflexion 120° | | | | | | | | | | | | | Bilat abduction 60° | | | | | | | | | | | Bilat abduction 120° | | | | | | | | | | |
|  |  | | CMC_w_ | | | CMC_b_ | | |  | | | | | CMC_w_ | | | CMC_b_ | | |  | | | | | | CMC_w_ | | | CMC_b_ | | | |  | | | | | CMC_w_ | | | | CMC_b_ | |  |
| *Controls dominant side* | | | | | | | | | | | | | | | | | | | | | | | | | | | | | | | | | | | | | | | | | | | | |
| Protraction | | **0.97** | | | **0.82** | | |  | | | | | **0.96** | | | | | **0.76** | | | |  | | | **0.78** | | | 0.44 | | | |  | | | | **0.92** | | | | **0.70** | | |  | |
| Lateral rotation | | **0.96** | | | **0.78** | | |  | | | | | **0.99** | | | | | **0.96** | | | |  | | | **0.96** | | | **0.89** | | | |  | | | | **0.99** | | | | **0.96** | | |  | |
| Tilt | | **0.87** | | | **0.62** | | |  | | | | | **0.91** | | | | | **0.66** | | | |  | | | **0.79** | | | 0.55 | | | |  | | | | **0.94** | | | | **0.75** | | |  | |
| *Controls non-dominant side* | | | | | | | | | | | | | | | | | | | | | | | | | | | | | | | | | | | | | | | | | | | | |
| Protraction | | **0.98** | | | **0.79** | | |  | | | | | **0.98** | | | | | **0.85** | | | |  | | | **0.80** | | | 0.53 | | | |  | | | | **0.84** | | | | 0.50 | | |  | |
| Lateral rotation | | **0.93** | | | **0.74** | | |  | | | | | **0.98** | | | | | **0.95** | | | |  | | | **0.97** | | | **0.81** | | | |  | | | | **0.99** | | | | **0.96** | | |  | |
| Tilt | | **0.77** | | | **0.70** | | |  | | | | | **0.92** | | | | | **0.61** | | | |  | | | **0.73** | | | **0.82** | | | |  | | | | **0.93** | | | | **0.75** | | |  | |
| *Stroke hemiplegic side* | | | | | | | | | | | | | | | | | | | | | | | | | | | | | | | | | | | | | | | | | | | | |
| Protraction | | **0.93** | | | **0.80** | | |  | | | | | **0.92** | | | | | **0.73** | | | |  | | | **0.82** | | | **0.61** | | | |  | | | | **0.94** | | | | **0.67** | | |  | |
| Lateral rotation | | **0.96** | | | **0.78** | | |  | | | | | **0.99** | | | | | **0.96** | | | |  | | | **0.92** | | | **0.84** | | | |  | | | | **0.99** | | | | **0.94** | | |  | |
| Tilt | | **0.94** | | | **0.77** | | |  | | | | | **0.97** | | | | | **0.83** | | | |  | | | **0.89** | | | 0.50 | | | |  | | | | **0.96** | | | | **0.75** | | |  | |
| *Stroke non-hemiplegic side* | | | | | | | | | | | | | | | | | | | | | | | | | | | | | | | | | | | | | | | | | | | | |
| Protraction | | **0.94** | | | **0.78** | | |  | | | | | **0.96** | | | | | **0.94** | | | |  | | | **0.83** | | | 0.37 | | | |  | | | | **0.86** | | | | 0.45 | | |  | |
| Lateral rotation | | **0.95** | | | **0.82** | | |  | | | | | **0.99** | | | | | **0.97** | | | |  | | | **0.94** | | | **0.85** | | | |  | | | | **0.99** | | | | **0.95** | | |  | |
| Tilt | | **0.94** | | | **0.75** | | |  | | | | | **0.93** | | | | | **0.86** | | | |  | | | **0.84** | | | 0.42 | | | |  | | | | **0.91** | | | | **0.68** | | |  | |
| CMC_w_ : adjusted coefficient of multiple correlation within session; CMC_b_ : adjusted coefficient of multiple correlation between sessions; Bilat: Bilateral; CMCs higher than 0.60 are marked in bold | | | | | | | | | | | | | | | | | | | | | | | | | | | | | | | | | | | | | | | | | | | | |
|  | |  | |  | | |  | | | |  | | | |  | | | |  | | | |  | | | |  | | | |  | | | |  | | | |  | | | |  | |
